# Identifying trauma patients with benefit from direct transportation to Level-1 trauma centers

**DOI:** 10.1186/s12873-021-00487-3

**Published:** 2021-08-06

**Authors:** Charlie A. Sewalt, Benjamin Y. Gravesteijn, Daan Nieboer, Ewout W. Steyerberg, Dennis Den Hartog, David Van Klaveren

**Affiliations:** 1 Department of Public Health, Erasmus MC University Medical Center, Na-building, room Na-2318, Wytemaweg 80, 3015 Rotterdam, CN The Netherlands; 2 Trauma Research Unit, Department of Surgery, Erasmus MC University Medical Center, Na-building, room Na-2318, Wytemaweg 80, 3015 Rotterdam, CN The Netherlands; 3 Department of Biomedical Data Sciences, Leiden University Medical Center, Leiden, The Netherlands

**Keywords:** Level 1 trauma center, Major trauma, Benefit

## Abstract

**Background:**

Prehospital triage protocols typically try to select patients with Injury Severity Score (ISS) above 15 for direct transportation to a Level-1 trauma center. However, ISS does not necessarily discriminate between patients who benefit from immediate care at Level-1 trauma centers. The aim of this study was to assess which patients benefit from direct transportation to Level-1 trauma centers.

**Methods:**

We used the American National Trauma Data Bank (NTDB), a retrospective observational cohort. All adult patients (ISS > 3) between 2015 and 2016 were included. Patients who were self-presenting or had isolated limb injury were excluded. We used logistic regression to assess the association of direct transportation to Level-1 trauma centers with in-hospital mortality adjusted for clinically relevant confounders. We used this model to define benefit as predicted probability of mortality associated with transportation to a non-Level-1 trauma center minus predicted probability associated with transportation to a Level-1 trauma center. We used a threshold of 1% as absolute benefit. Potential interaction terms with transportation to Level-1 trauma centers were included in a penalized logistic regression model to study which patients benefit.

**Results:**

We included 388,845 trauma patients from 232 Level-1 centers and 429 Level-2/3 centers. A small beneficial effect was found for direct transportation to Level-1 trauma centers (adjusted Odds Ratio: 0.96, 95% Confidence Interval: 0.92–0.99) which disappeared when comparing Level-1 and 2 versus Level-3 trauma centers. In the risk approach, predicted benefit ranged between 0 and 1%. When allowing for interactions, 7% of the patients (*n* = 27,753) had more than 1% absolute benefit from direct transportation to Level-1 trauma centers. These patients had higher AIS Head and Thorax scores, lower GCS and lower SBP. A quarter of the patients with ISS > 15 were predicted to benefit from transportation to Level-1 centers (*n* = 26,522, 22%).

**Conclusions:**

Benefit of transportation to a Level-1 trauma centers is quite heterogeneous across patients and the difference between Level-1 and Level-2 trauma centers is small. In particular, patients with head injury and signs of shock may benefit from care in a Level-1 trauma center. Future prehospital triage models should incorporate more complete risk profiles.

**Supplementary Information:**

The online version contains supplementary material available at 10.1186/s12873-021-00487-3.

## Background

In western countries, injury is the major cause of death in adults younger than 45 years of age [1]. Over the years, systems of trauma care have been developed including the introduction of Level-1 trauma centers [2]. Level-1 trauma centers are hospitals which are fully equipped to care for severely injured patients [2]. In the United States, Level-1 and Level-2 trauma centers are considered higher-level trauma centers and Level-3 and 4 are considered lower-level trauma centers (Table 1) [3]. Pre-hospital triage, aiming to identify trauma patients at-risk for worse outcome, is necessary to provide early care while transporting the patient to the appropriate hospital with needed facilities [4, 5].

**Table 1:** Trauma center levels by the American College of Surgeons (ACS)

	Level-1	Level-2	Level-3	Level-4
Goal	Total care of every aspect of injury	Definitive care for all injured patients	Prompt assessment, resuscitation, surgery, intensive care and stabilization of injured patients and emergency operations.	Provide ATLS prior to transfer patients to higher level trauma center
Coverage	24 h of all specialties	24 h surgeons, neurosurgery, anaesthesiology, emergency medicine and radiology	24 h emergency medicine	Basic emergency department facilities
Volume	Minimum annual volume of severely injured patients	No minimum annual volume of severely injured patients	No minimum annual volume of severely injured patients	No minimum annual volume of severely injured patients

Globally, there is consensus that patients with major trauma, most often defined as an Injury Severity Score (ISS) above 15, could benefit from direct transportation to Level-1 trauma centers [3]. Therefore, prehospital triage protocols try to select patients with an ISS above 15 for direct transportation to a Level-1 trauma center [6]. However, ISS might not necessarily discriminate between patients who benefit from transportation to a Level-1 trauma center and those who do not benefit from transportation to a Level-1 trauma center. Due to under triage, trauma patients are often transferred to a non-trauma center, often followed by a delayed transfer to a Level-1 trauma center. Only half of these secondary transferred patients are having an ISS above 15 which shows that more trauma patients might benefit from direct transportation to Level-1 trauma centers [7]. The delay in optimal treatment is potentially harmful and leads to increased mortality and morbidity [8]. Also, most American prehospital triage protocols do not distinguish between Level-1 trauma centers and Level-2 trauma centers.

To enable admission of trauma patients to the right hospital as quickly as possible and to make optimal use of resources, we aimed to assess which trauma patients benefit from direct transportation to a Level-1 trauma center. Also, we assessed which patients benefit from direct transportation to a Level-1 or Level-2 trauma center.

## Methods

### Study design

We used data from the American National Trauma Data Bank (NTDB), a retrospective observational cohort study in the United States. NTDB is the largest trauma registry in the United States and provides patient demographics, information about vital parameters, interventions received and in-hospital outcomes [9].

### Participants

All injured adults (> 18 years) included in NTDB between January 1, 2015 and December 31, 2016 and treated in a Level-1/2/3 trauma center (on both the ACS level and state level) were included. We did not include patients treated in state Level-4 trauma centers, because differences in case-mix compared to the other trauma centers were too large. We selected patients in whom it is likely that the decision of transportation to a level-1 trauma center likely affects outcome. Hereto, we used exclusion criteria partly based on the Victoria Falls trauma registry and Trauma Audit and Research Network [10]:

- Isolated limb injury (defined as AIS extremity 1 to 5 and other AIS categories equal to 0)
- Duplicates
- Not transported with ambulance or helicopter
- ISS below 4
- Children (age below 18 years)
- No vital signs at arrival

### Data preparation

ACS level designation was extracted and for centers without ACS level designation, their state level designation was used. Centers without level designation or state Level-4 designation were excluded. The abbreviated injury scales were extracted and combined [11]: head and neck AIS were combined into head/neck AIS variable; the upper and lower extremity AIS were combined into limb AIS variable; the skin and unspecified AIS scores were combined into the other AIS variable. For prehospital vital signs, the maximum value registered in the ambulance was used. The Charlson comorbidity index was calculated from the individual reported chronic diseases [12]. For intubation, the following ICD10 procedure codes were used: 0BH17EZ, 0BH18EZ, 5A1945Z, 0WH30YZ, 5A09357, Z99.89 and 5A09358. Death was defined as “Deceased/expired” or “discharged/transferred to hospice care”. When the outcome was reported as “Left against medical advice or discontinued care”, “Not applicable”, or “Not known/not recorded”, hospital outcome was considered missing. Finally, transportation to a Level-1 trauma center was defined by the first initial transport: patients who were transferred from a Level-2/3 facility to a Level-1 facility were considered a non-Level-1 trauma center patient.

### Statistical analysis

Patients transported to Level-1 trauma centers were compared to patients transported to Level-2 and Level-3 trauma centers using descriptive statistics: median and interquartile range (IQR) for the continuous variables, and frequency and percentage of the total group for categorical variables. Continuous variables were compared using the Kruskal-Wallis test, and categorical variables using the chi-square test. To include all patients in the main analysis, we imputed the dataset using the *Hmisc* package in R, assuming missingness at random [13].

The association between in-hospital mortality and transportation to a Level-1 trauma center rather than a Level-2/3 trauma center was assessed using logistic regression. We first fitted a unadjusted model with only the effect of Level-1 versus Level-2/3 trauma centers. Second we adjusted for clinically relevant confounders: age; sex; Charlson comorbidity index, prehospital physiological parameters (saturation, systolic blood pressure (SBP), respiration rate (RR), pulse, Glasgow Coma Score (GCS), AIS Head, AIS Thorax, AIS Abdomen, AIS Extremity and AIS Spine. The continuous variables age, saturation, pulse, systolic blood pressure (SBP) and respiration rate (RR) were included as restricted cubic spline to allow for non-linearity [14]. Similarly, the ordinal variables GCS, AIS Head, AIS Thorax, AIS Abdomen, AIS Extremity and AIS Spine were included as quadratic polynomials (x + x^2^). Third, we included interactions of transportation to a level-1 center with five baseline characteristics which we hypothesized to potentially modify the benefit from treatment in Level-1 trauma centers: AIS Head, AIS Thorax, AIS Abdomen, systolic blood pressure (SBP) and Charlson comorbidity index. In order to avoid overfitting, a maximum likelihood penalized logistic regression model was used [15].

In order to predict the benefit of transportation to a Level-1 trauma center, the predicted probability of mortality associated with transportation to a Level-1 trauma center was subtracted from the predicted probability of mortality associated with transportation to a Level-2/3 trauma center [16] for both the pure risk model and the model including interaction terms. We plotted the observed benefit versus the predicted benefit in 20 categories of predicted benefit to assess calibration for benefit. A threshold of more than 1% absolute predicted benefit was used as definition of benefit. A predicted absolute benefit of 1% for a particular patient means 1% less mortality when this particular patient is brought to a Level-1 trauma center instead of a Level-2/3 trauma center. A threshold of more than 1% absolute predicted harm was used as definition of harm. To assess the differences between ISS > 15 and Benefit > 1%, a cross table was made and observed benefit was calculated (mortality rate in Level-2/3 trauma centers minus mortality rate in Level-1 trauma centers for each bin of the cross table). Observed benefit above 0% means less mortality patients transported to Level-1 trauma centers and observed benefit below 0% means more mortality for patients transported to Level-1 trauma centers.

The same analysis mentioned above was repeated comparing patients directly transported to a Level-1 or Level-2 trauma center compared to patients transported to a Level-3 trauma center. The same potential confounders and models were used as mentioned above.

Finally, as a sensitivity analysis, we used bootstrapping to identify patients for whom the probability of a positive predicted benefit exceeded 95%. Hereto, 1000 replicates of the coefficients were drawn from a multivariate normal distribution, and these were used to obtain predictions for the patients. A patient was considered to have a substantial benefit if the predicted benefit was above zero in at least 950/1000 (equal to 5% significance) of the replications.

All analyses were repeated without imputation. All analyses were performed using R version 3.6.1 (R Core Team (2013): A language and environment for statistical computing. R Foundation for Statistical Computing, Vienna, Austria). This study was reported conform the STROBE guidelines [17].

## Results

### Descriptives

**Fig. 1:**
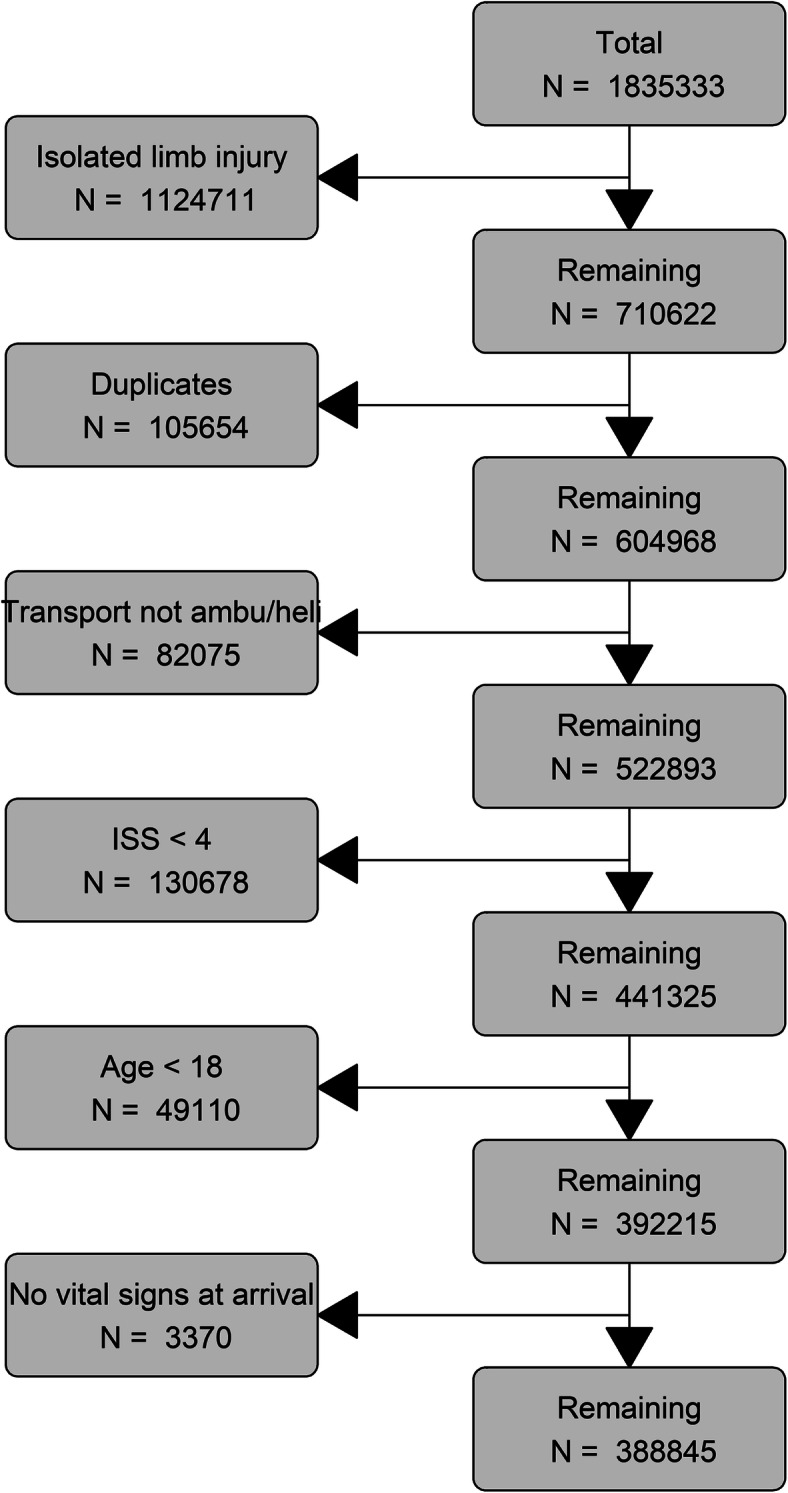
flowchart of the study

A total of 388,845 trauma patients from 232 Level-1 trauma centers (*n* = 226,640) and 429 Level-2/3 trauma centers (*n* = 162,205) were included (Fig. 1). Patients transported to Level-1 trauma centers were more severely injured compared to patients transported to Level-2 and Level-3 trauma centers (ISS > 15 34% compared to 28.4 and 20.4% respectively). Also, they were more often comatose (8.7% compared to 6.8 and 4.9% respectively) and intubated (4.5% compared to 4.0 and 1.8% respectively). Travel time to the first hospital varied from 52 min (IQR 36–76) for patients transported to Level-1 trauma centers to 42 min (IQR 33–57) for patients transported to Level-3 trauma centers. Missingness of the variables was comparable across the different levels of trauma centers (Table 1). All differences were statistically significant (*p* < 0.001, Table 2).

**Table 2:** Baseline characteristics for Level-1/2/3 Trauma Centers

	Level-1 Trauma Centers	Level-2 Trauma Centers	Level-3 Trauma Centers	Missing
N	226,640	137,398	24,807	
Age (median [IQR])	51 [32, 68]	54 [34, 71]	58 [37, 75]	0.0
Male (%)	150,334 (66.3)	87,838 (63.9)	14,719 (59.3)	0.0
Charlson comorbidty index > 2	6189 (2.7)	4181 (3.0)	945 (3.8)	0.0
ISS (median [IQR])	10 [6, 17]	10 [5, 17]	9 [5, 14]	0.0
ISS > 15 (%)	77,001 (34.0)	39,054 (28.4)	5150 (20.8)	0.0
AIS Head > 2 (%)	63,055 (27.8)	36,000 (26.2)	5264 (21.2)	0.0
AIS Thorax > 2 (%)	55,170 (24.3)	29,675 (21.6)	4736 (19.1)	0.0
AIS Face > 2 (%)	2391 (1.1)	1103 (0.8)	152 (0.6)	0.0
AIS Abdomen > 2 (%)	16,388 (7.2)	7422 (5.4)	1015 (4.1)	0.0
AIS Spine > 2 (%)	16,714 (7.4)	9229 (6.7)	1382 (5.6)	0.0
AIS Extremity > 2 (%)	26,714 (11.8)	14,179 (10.3)	2355 (9.5)	0.0
AIS Other > 2 (%)	1000 (0.4)	297 (0.2)	73 (0.3)	0.0
Prehospital features				
Oxygen saturation (median [IQR])	98 [95, 99]	97 [95, 99]	97 [95, 98]	45.4
Respiration rate (median [IQR])	18 [16, 20]	18 [16, 20]	18 [16, 20]	35.3
Pulse (median [IQR])	90 [78, 104]	89 [77, 102]	88 [76, 100]	33.6
Systolic blood pressure (median [IQR])	136 [118, 153]	138 [120, 156]	140 [122, 158]	34.2
Hypotension (SBP < 90) (%)	6291 (4.7)	4172 (4.1)	691 (3.5)	34.2
Glasgow Coma Score (median [IQR])	15 [15, 15]	15 [15, 15]	15 [15, 15]	1.5
Comatose (GCS < 8) (%)	19,352 (8.7)	9251 (6.8)	1192 (4.9)	1.5
Intubated (%)	10,233 (4.5)	5529 (4.0)	457 (1.8)	0.1
Travel time to first hospital (median [IQR])	52 [36, 76]	47 [34, 67]	42 [33, 57]	24.4
Outcome measures				
Transferred from other hospital (%)	87,684 (38.7)	35,335 (25.7)	2438 (9.8)	0.0
Length of stay (median [IQR])	4 [2, 8]	4 [2, 7]	3 [1, 5]	2.3
In-hospital mortality (%)	12,890 (6.1)	7265 (5.7)	808 (4.5)	8.1

### Main effect of Level-1 trauma centers on in-hospital mortality

Without adjustment for baseline characteristics, transportation to a Level-1 trauma center was associated with higher in-hospital mortality (OR: 1.09, 95% CI: 1.06–1.12). After adjustment for clinically relevant variables, we found a small significant beneficial effect of transportation to a Level-1 trauma center on in-hospital mortality (OR: 0.96, 95% CI: 0.92–0.99).

### Benefit from Level-1 trauma centers

**Fig. 2:**
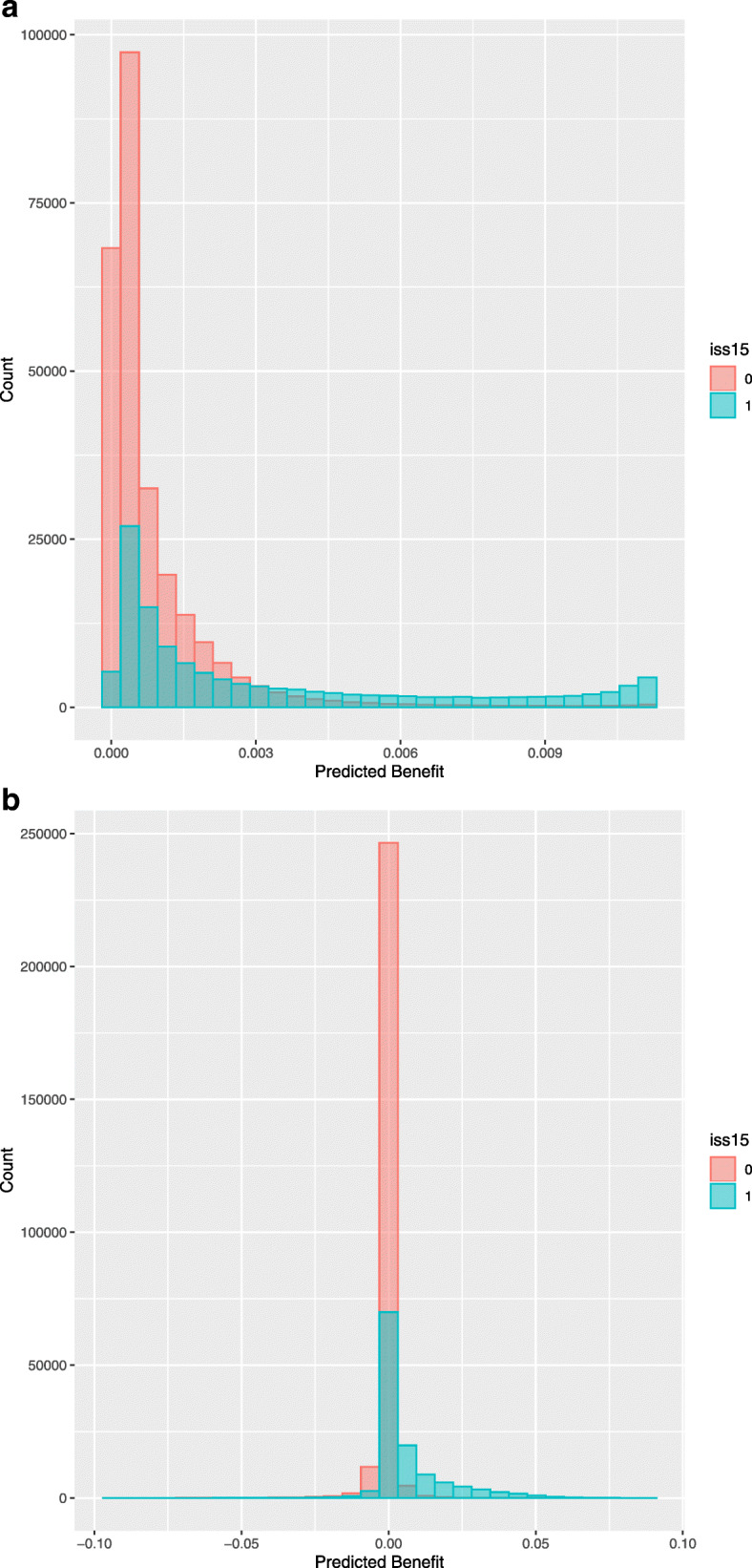
Histograms of the range of absolute benefit, for both the model with (**B**) and without interaction terms (**A**). Both histograms show the difference in absolute benefit for patients with ISS above 15 and patients with ISS below 15. A predicted absolute benefit of 1% (0.01) for a particular patient means 1% less mortality when this particular patient is brought to a Level-1 trauma center instead of a Level-2/3 trauma center

**Fig. 3:**
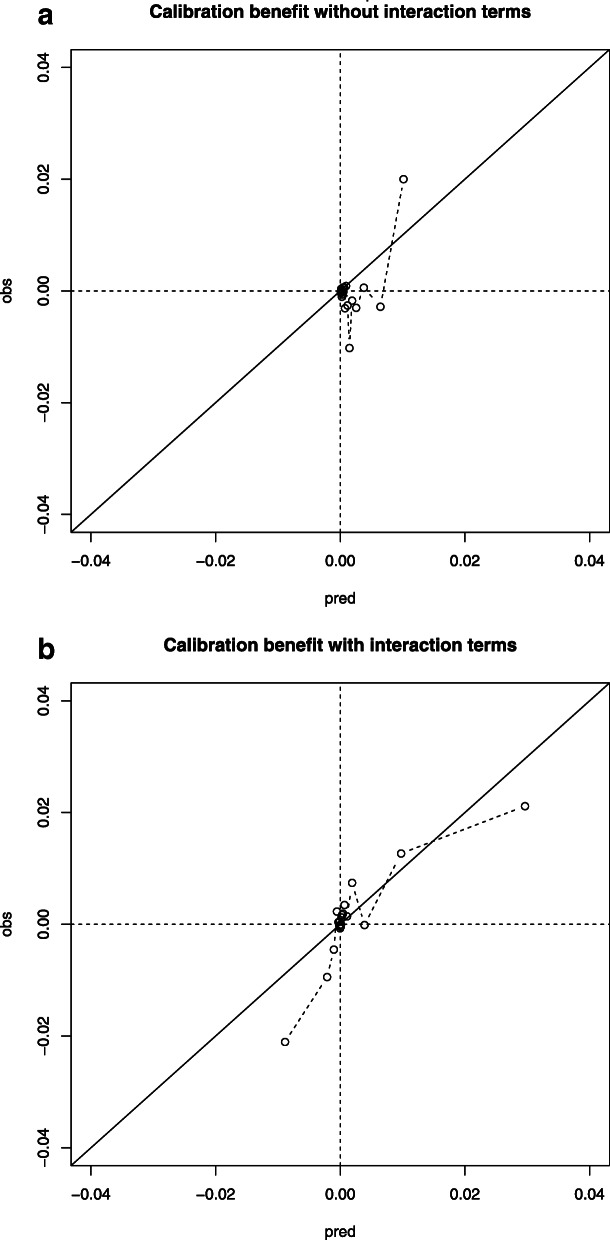
Calibration of benefit, for both the model with (**B**) and without interaction terms (**A**). Calibration of benefit is plotted with the predicted absolute treatment benefit on the x-axis and observed absolute treatment benefit on the y-axis. The study population was therefore divided in 20 equal groups based on the predicted absolute treatment benefit. A predicted absolute benefit of 1% (0.01) for a particular patient means 1% less mortality when this particular patient is brought to a Level-1 trauma center instead of a Level-2/3 trauma center

In the pure risk approach without interaction terms, all patients were calculated to substantially benefit from treatment in Level-1 trauma centers, but the absolute benefit was limited to a maximum of 1%. (Fig. 2). Based on clinical relevance, the following interaction terms were added to the model: AIS Head, AIS Abdomen, AIS Thorax, prehospital SBP and Charlson Comorbidity Index. These effects were very subtle compared to the main effects (Table S1). For the model including interaction terms, 27,753 patients (7%) had a predicted benefit above 1% from transportation to a Level-1 trauma center (Fig. 2). The concordance between expected and observed benefit was moderate when interaction terms were included (Fig. 3). Patients who were predicted to have more than 1% absolute benefit following the model with interaction terms had higher AIS Head and AIS Thorax scores, lower GCS scores and lower systolic blood pressures (Table 3). Of all patients with predicted benefit, 17,739 (63.9%) were currently directly transported to a Level-1 trauma center. A quarter of the patients with ISS > 15 were predicted to benefit from transportation to Level-1 centers (*n* = 26,522, 22%). Of these 26,522 patients, the observed benefit was 1.4% meaning that patients transported to Level-1 trauma centers had 1.4% lower mortality. The observed benefit for patients with an ISS < 15 but predicted benefit > 1% was 6.4% (*n* = 1231). The observed benefit of patients with an ISS > 15 but predicted benefit < 1% was − 22% meaning that this group of patients had 22% higher mortality when being transported to a Level-1 trauma center instead of a Level-2/3 trauma center (*n* = 94,683).

In the approach where patients were identified who were predicted to substantially benefit from transportation to a Level-1 trauma center (95% CI lower bound of benefit above zero when considering the model with interaction terms), we found a larger group of patients with benefit (91,758). Nevertheless, similar characteristics differed between the benefit and no benefit group: patients with substantial benefit had higher AIS Thorax, had a higher AIS Head, lower GCS and lower SBP (Table S3). ISS was higher than 15 in 60,092 patients (65.6%) in the predicted benefit group, but 61,113 patients considered severely injured (ISS > 15: 50.4%) were in the group without predicted benefit from transportation to Level-1 trauma centers (Figure S3).

#### Harm from Level-1 trauma centers

Four thousand five hundred twenty-five patients (1%) were predicted to be harmed from direct transportation to Level-1 trauma centers. These patients were older and had more serious comorbidities (Table 3).

**Table 3:** Characteristics of patients who benefit from transportation to a Level-1 trauma centers, based on predicted absolute benefit from the regression model with interaction terms. Harm was defined as more than 1% absolute harm, while benefit was defined as more than 1% absolute benefit

	Harm	No benefit/harm	Benefit
N	4525	361,092	27,753
Age (median [IQR])	79 [71, 85]	53 [33, 69]	53 [32, 71]
Male (%)	2717 (60.0)	232,812 (64.5)	20,117 (72.5)
Charlson Comorbidity Index > 1 (%)	3917 (86.6)	29,518 (8.2)	1042 (3.8)
ISS (median [IQR])	10 [5, 16]	10 [5, 16]	27 [25, 35]
ISS > 15 (%)	1308 (28.9)	94,683 (26.2)	26,522 (95.6)
AIS Head > 3 (%)	701 (15.5)	30,755 (8.5)	20,191 (72.8)
AIS Thorax > 3 (%)	108 (2.4)	10,775 (3.0)	4163 (15.0)
AIS Face > 3 (%)	5 (0.1)	224 (0.1)	220 (0.8)
AIS Abdomen > 3 (%)	41 (0.9)	7668 (2.1)	2606 (9.4)
AIS Spine > 3 (%)	156 (3.4)	5718 (1.6)	926 (3.3)
AIS Extremity > 3 (%)	32 (0.7)	3041 (0.8)	969 (3.5)
AIS Other > 3 (%)	30 (0.7)	734 (0.2)	66 (0.2)
**Prehospital features**			
Oxygen saturation (median [IQR])	96 [93, 98]	98 [95, 99]	96 [90, 98]
Respiration Rate (median [IQR])	18 [16, 20]	18 [16, 20]	16 [12, 20]
Pulse (median [IQR])	85 [73, 100]	89 [77, 102]	90 [74, 110]
SBP (median [IQR])	162 [138, 189]	138 [121, 156]	121 [96, 141]
SBP < 90 (%)	48 (1.1)	11,487 (3.2)	5258 (18.9)
GCS (median [IQR])	15 [13, 15]	15 [15, 15]	6 [3, 13]
GCS < 8 (%)	667 (14.7)	12,774 (3.5)	17,428 (62.8)
Intubated (%)	311 (6.9)	12,316 (3.4)	3945 (14.2)
Travel time to first hospital (median [IQR])	52 [39, 76]	49 [35, 71]	48 [32, 76]
**Outcome measures**			
Transferred from other hospital (%)	1770 (39.1)	116,755 (32.3)	8708 (31.4)
Level trauma center (%)			
1	2414 (53.3)	208,901 (57.9)	17,739 (63.9)
2	1747 (38.6)	128,501 (35.6)	8897 (32.1)
3	364 (8.0)	23,690 (6.6)	1117 (4.0)
Length of stay (median [IQR])	5 [3, 9]	4 [2, 7]	8 [2, 19]
In-hospital mortality (%)	1018 (22.5)	11,992 (3.3)	10,759 (38.8)

### Main effect of Level-1/2 trauma centers on in-hospital mortality

Without adjustment for baseline characteristics, transportation to a Level-1 or Level-2 trauma center was associated with higher in-hospital mortality (OR: 1.25, 95% CI: 1.18–1.33). After adjustment for clinically relevant variables, we found no significant effect of transportation to a Level-1 or Level-2 trauma center on in-hospital mortality (OR: 0.98, 95% CI: 0.92–1.06).

### Benefit from Level-1/2 trauma centers

In the pure risk approach without interaction terms, all patients were calculated to substantially benefit from treatment in Level-1 or Level-2 trauma centers, but the absolute benefit was limited to a maximum of 0.5%. (Figure S2). For the model including interaction terms, 31,517 patients had a predicted benefit above 1% from transportation to a Level-1 or Level-2 trauma center (Figure S2). The concordance between expected and observed benefit was moderate when interaction terms were included (Figure S1). Patients who were predicted to have more than 1% absolute benefit following the model with interaction terms had higher AIS Head and AIS Thorax scores, lower GCS scores and lower systolic blood pressures (Table S2). Of all patients with predicted benefit, 19,793 (62.8%) were currently directly transported to a Level-1 trauma center and 10,364 (32.9%) patients were currently transported to a Level-2 trauma center. 10.764 patients (2.7%) were predicted to be harmed from direct transportation to Level-1 or Level-2 trauma centers. These patients were older and had more serious comorbidities (Table S2). A quarter of the patients with ISS > 15 were predicted to benefit from transportation to Level-1 or Level-2 trauma centers (*n* = 29,441, 24%).

All analyses were repeated with the raw data without imputation and showed similar results.

## Discussion

This study aimed to assess which trauma patients benefit from direct transportation to Level-1 trauma centers. The benefit of transportation to a Level-1 trauma centers is quite heterogeneous across patients and may be limited overall. The existence of an overall beneficial effect of Level-1 trauma centers justifies the search for specific subgroups of trauma patients having most predicted benefit. Benefit of Level-1 trauma centers does not only increase with higher ISS, reflecting more severely injured patients with high risk of mortality, but is also attributable to individual patient characteristics. In particular, head injury, thorax injury and signs of shock were observed in patients who could benefit from treatment in Level-1 trauma centers. When comparing Level-1/2 trauma centers with Level-3 trauma centers, no overall beneficial effect on mortality was found.

Our study shows that benefit of transportation to a Level-1 trauma center is attributable to patient characteristics and that patients with an ISS < 15 but predicted benefit above 1% have a observed survival benefit when being transported to a Level-1 trauma center. Also, we found that only a quarter of the patients with ISS > 15 have a predicted benefit above 1% when being transported to a Level-1 trauma center. These results questions the use of solely ISS as definition of severe injury and consequently for selecting level of trauma center. Over the years, ISS > 15 as only indicator of severe injury is more and more subject of debate. First, an equal Abbreviated Injury Scale (AIS) in different body regions is assumed to be equal in injury severity [18, 19]. Second, ISS does not account for multiple injuries in the same body region [19, 20]. So it is possible that patients with equal ISS scores do not have the same injury severity. Third, ISS does not take other important predictors of in-hospital mortality and potential benefit from Level-1 trauma centers into account, like physiological parameters, age and comorbidities. Using solely ISS > 15 as definition of severe injury and consequently for selecting level of trauma center could result in large overtriage ratios (up to 78%).

With the ageing population it is important to assess the effect of frailty, for example by looking at age and comorbidities, on potential benefit of direct transportation to Level-1 trauma centers [21, 22]. We found that patients with higher age and more serious comorbidities benefit less from direct transportation to Level-1 trauma centers and some elderly patients might even be harmed from direct transportation to Level-1 trauma centers. These results might seem surprising at first, but elderly patients with comorbidities have less potential benefit from direct transportation to Level-1 trauma centers since specialized treatment might not be applicable or desirable in this group of patients and could cause more serious complications [23, 24].

We also assessed direct transportation to Level-1 or Level-2 trauma centers compared to Level-3 trauma centers, because Level-2 trauma centers are close in capability to Level-1 trauma centers. We found comparable results showing that head injury, thorax injury and signs of shock were observed in patients who could benefit from treatment in Level-1 or Level-2 trauma centers. There was no overall difference in outcome between Level-1 and Level-2 trauma centers. Previous research shows that patients with severe Traumatic Brain Injury and major trauma have better outcomes when being transported to Level-1 trauma centers instead of Level-2 trauma centers [3, 25]. We did find an overall beneficial effect of Level-1 trauma centers. Our study indicates that patients with major trauma in terms of head injury and signs of shock might have benefit from direct transportation to Level-1 trauma centers over Level-2 trauma centers.

Present study shows that benefit of Level-1 trauma centers does not only increase with higher ISS reflecting more severely injured patients with high risk of mortality, but is also dependent on individual patient characteristics. Selecting patients on variables corresponding with predicted benefit is an important addition to trying to select patient with ISS above 15 only. This could improve triage and therefore immediate admittance of trauma patients to the right level of center. Our study shows that a general trauma population could already benefit from treatment in Level-1 trauma centers. Previous studies found comparable results for Level-1 trauma centers [2, 3]. Our study adds that prehospital triage for trauma patients should not only focus on the overall risk of mortality, but also on individual patient characteristics which indicate benefit from transportation to a Level-1 trauma center. According to our study, patients with head injury, thorax injury and signs of shock should be directly transported to Level-1 trauma centers to improve survival. This was in accordance with an American study, which also found benefit from Level-1 trauma centers for head injury patients [26].

### Strengths

Our study has several strengths. First, we have used a retrospective observational cohort study, the American National Trauma Data Bank (NTDB) which gave us a large sample size with many potential predictors of benefit from Level-1 trauma centers. Second, we used a statistical approach which made it possible to calculate the benefit of treatment in a Level-1 trauma center for each individual patient.

### Limitations

This study has several limitations too. First, we had to deal with missing data. Especially for prehospital measured physiological parameters, like saturation or systolic blood pressure, approximately 35% was missing. We dealt with this by using multiple imputation, a method proven to give valid estimates under the missing at random assumption [27]. Second, we used observational data which makes it difficult to look at causality. Since part of the patients is transported between hospitals, which makes it difficult to obtain the effect of individual predictors and therefore more research is needed to validate our results. Third, we used significant and clinically relevant interaction terms to predict benefit and calculated both the absolute benefit above 1% and the 95% CI of benefit to assess our ‘substantial’ benefit group. Both methods are not entirely satisfying since using 1% as threshold for benefit is an arbitrary cut-off and using substantial ‘significant’ benefit does not distinguish between neglectable benefit and impactful benefit. However since both approaches provide us with comparable results we do think that our results are valid.

### Implications

Our study is a first step towards a prehospital triage protocol based on benefit from level of trauma care. Physiological parameters are usable in triaging trauma patients to the right hospital, but their predictive utilities in major trauma patients are not ideal [28]. We found that only systolic blood pressure (as sign of shock) and signs of head injury were providing information about having benefit from Level-1 trauma centers. About one-third of the patients without predicted benefit from Level-1 trauma centers is still transferred to a Level-1 trauma center. Therefore, a prehospital triage protocol should include a combination of both physiological parameters, injury features and patient-related factors (i.e. comorbidity). Patient groups that might experience benefit from Level-1 trauma centers might differ across countries and trauma systems. This makes it necessary to develop a country specific approach to assess benefit from Level-1 trauma centers in order to minimize inter-hospital transfers and maximize patient outcomes.

## Conclusions

Benefit of transportation to a Level-1 trauma centers is quite heterogeneous across patients and the difference between Level-1 and Level-2 trauma centers is small. Benefit of Level-1 trauma centers does not only increase with higher ISS reflecting more severely injured patients with high risk of mortality, but is also dependent on individual patient characteristics. In particular, head injury, thorax injury and signs of shock were observed in patients who could benefit from treatment in Level-1 trauma centers. Further research should focus on identifying these patients based on simple prehospital baseline characteristics in order to bring the right patient to the right hospital. Selecting patients on variables corresponding with predicted benefit is an important addition which could improve triage and therefore immediate admittance of trauma patients to the right level of center.

## Supplementary Information

**Additional file 1: Table S1.** Odds ratios with 95% confidence interval for the three regression models calculating benefit from direct transportation to Level-1 trauma center. For continuous variables the odds ratio of the 75th percentile versus the median and median versus 25th percentile is presented. Continuous variables from interaction terms are centered in order to make the main effects interpretable. Odds ratios above 1 indicate an increase in the probability of mortality. **Table S2.** Characteristics of patients who benefit from transportation to a Level-1/2 trauma centers, based on predicted absolute benefit from the regression model with interaction terms. Harm was defined as more than 1% absolute harm, while benefit was defined as more than 1% absolute benefit. **Table S3.** Characteristics of patients who substantially benefit from transportation to a Level-1 trauma centers, based on predicted benefit above 0 in 95% of bootstrapped samples from the regression model with interaction terms.

**Additional file 2: Figure S1.** Calibration of benefit, for both the model with (B) and without interaction terms (A). Calibration of benefit is plotted with the predicted absolute treatment benefit on the x-axis and observed absolute treatment benefit on the y-axis. The study population was therefore divided in 20 equal groups based on the predicted absolute treatment benefit. A predicted absolute benefit of 1% (0.01) for a particular patient means 1% less mortality when this particular patient is brought to a Level-1/2 trauma center instead of a Level-3 trauma center.

**Additional file 3: Figure S2.** Histograms of the range of absolute benefit, for both the model with (B) and without interaction terms (A). Both histograms show the difference in absolute benefit for patients with ISS above 15 and patients with ISS below 15. A predicted absolute benefit of 1% (0.01) for a particular patient means 1% less mortality when this particular patient is brought to a Level-1/2 trauma center instead of a Level-3 trauma center.

**Additional file 4: Figure S3.** Patients who benefit with predicted benefit above 0 in 95% of the predictions from transportation to a Level-1 trauma center, following the models without (A) and with interaction terms (B).

## Data Availability

The datasets analysed during the current study are available at https://www.facs.org/quality-programs/trauma/tqp/center-programs/ntdb/datasets. The R code can be made available upon request.

## Abbreviations

ACS: American College of Surgeons
AIS: Abbreviated Injury Scale
GCS: Glasgow Coma Score
IQR: Interquartile Range
ISS: Injury Severity Score
NTDB: National Trauma Data Bank
RR: Respiration Rate
SBP: Systolic Blood Pressure
