# Supplementary material for: Identifying trauma patients with benefit from direct transportation to Level-1 trauma centers
Source: BMC Emerg Med. 2021 Aug 6;21:93. doi: 10.1186/s12873-021-00487-3 (PMC8344140; doi:10.1186/s12873-021-00487-3)
Supplement: Supplementary file 1 — Additional file 1: Table S1. Odds ratios with 95% confidence interval for the three regression models calculating benefit from direct transportation to Level-1 trauma center. For continuous variables the odds ratio of the 75th percentile versus the median and median versus 25th percentile is presented. Continuous variables from interaction terms are centered in order to make the main effects interpretable. Odds ratios above 1 indicate an increase in the probability of mortality. Table S2. Characteristics of patients who benefit from transportation to a Level-1/2 trauma centers, based on predicted absolute benefit from the regression model with interaction terms. Harm was defined as more than 1% absolute harm, while benefit was defined as more than 1% absolute benefit. Table S3. Characteristics of patients who substantially benefit from transportation to a Level-1 trauma centers, based on predicted benefit above 0 in 95% of bootstrapped samples from the regression model with interaction terms. [file 12873_2021_487_MOESM1_ESM.docx]

**Supplementary material**

Table S1: Odds ratios with 95% confidence interval for the three regression models calculating benefit from direct transportation to Level-1 trauma center. For continuous variables the odds ratio of the 75^th^ percentile versus the median and median versus 25^th^ percentile is presented. Continuous variables from interaction terms are centered in order to make the main effects interpretable. Odds ratios above 1 indicate an increase in the probability of mortality.

| OR (95% CI) | **Model 1: unadjusted** | **Model 2: adjusted without interaction terms** | **Model 3: adjusted with interaction terms (unpenalized)** | **Model 3: adjusted with interaction terms**  **(penalized)** |
| --- | --- | --- | --- | --- |
| Level-1 versus Level-2/3 | 1.09 (1.06-1.12) | 0.96 (0.92-0.99) | 0.98 (0.94-1.02) | 0.98 (0.94-1.02) |
| Charlson comorbidity index  1 vs 0 | - | 1.32 (1.30-1.34) | 1.26 (1.24-1.28) | 1.26 (1.24-1.28) |
| Male | - | 1.29 (1.24-1.33) | 1.29 (1.24-1.33) | 1.29 (1.24-1.33) |
| Age 53 vs 33 | - | 1.43 (1.37-1.51) | 1.43 (1.36-1.50) | 1.43 (1.36-1.50) |
| Age 70 vs 53 | - | 1.63 (1.55-1.70) | 1.63 (1.56-1.71) | 1.63 (1.56-1.71) |
| Oxygen saturation 97 vs 95 | - | 0.96 (0.95-0.97) | 0.96 (0.96-0.97) | 0.96 (0.96-0.97) |
| Oxygen saturation 99 vs 97 | - | 0.94 (0.93-0.95) | 0.94 (0.93-0.96) | 0.94 (0.93-0.96) |
| Respiration Rate  18 vs 16 | - | 0.94 (0.93-0.95) | 0.94 (0.93-0.95) | 0.94 (0.93-0.95) |
| Respiration Rate  20 vs 18 | - | 1.09 (1.08.1.10) | 1.09 (1.08-1.11) | 1.09 (1.08-1.11) |
| Pulse  89 vs 77 | - | 0.93 (0.91-0.95) | 0.93 (0.91-0.95) | 0.93 (0.91-0.95) |
| Pulse  102 vs 89 | - | 1.17 (1.14-1.20) | 1.17 (1.14-1.20) | 1.17 (1.14-1.20) |
| Systolic blood pressure  137 vs 120 | - | 0.83 (0.82-0.84) | 0.70 (0.67-0.71) | 0.70 (0.67-0.71) |
| Systolic blood pressure  155 vs 137 | - | 1.19 (1.17-1.21) | 1.34 (1.30-1.38) | 1.34 (1.30-1.38) |
| GCS 15 vs 14 | - | 0.99 (0.98-1) | 0.99 (0.98-0.99) | 0.99 (0.99-0.99) |
| AIS Head 2 vs 0 | - | 1.46 (1.45-1.48) | 1.62 (1.60-1.65) | 1.62 (1.60-1.65) |
| AIS Thorax 1 vs 0 | - | 1.11 (1.10-1.12) | 1.21 (1.19-1.24) | 1.21 (1.19-1.24) |
| AIS Abdomen 1 vs 0 | - | 1.09 (1.08-1.10) | 1.09 (1.07-1.10) | 1.09 (1.07-1.10) |
| AIS Limb 2 vs 0 | - | 1.23 (1.20-1.26) | 1.23 (1.20-1.26) | 1.23 (1.20-1.26) |
| AIS Spine 2 vs 0 | - | 1.23 (1.21-1.25) | 1.23 (1.20-1.25) | 1.23 (1.20-1.25) |
| Level*AIS Head | - | - | 0.95 (0.93-0.98) | 0.95 (0.93-0.98) |
| Level*AIS Abdomen | - | - | 0.99 (0.96-1.02) | 0.99 (0.96-1.02) |
| Level*AIS Thorax | - | - | 0.98 (0.95-1.02) | 0.98 (0.95-1.02) |
| Level*Charlson comorbidity index | - | - | 1.02 (1-1.05) | 1.02 (1-1.05) |
| Level*SBP | - | - | 1.04 (1.01-1.07) | 1.04 (1.01-1.07) |

Table S2: Characteristics of patients who benefit from transportation to a Level-1/2 trauma centers, based on predicted absolute benefit from the regression model with interaction terms. Harm was defined as more than 1% absolute harm, while benefit was defined as more than 1% absolute benefit.

|  | | Harm | No benefit/harm | Benefit |
| --- | --- | --- | --- | --- |
| N | | 10,764 | 346,564 | 31,517 |
| Age (median [IQR]) | | 79 [65 , 85 ] | 52 [32 , 68 ] | 57 [35 , 76 ] |
| Male (%) | | 6740 (62.6) | 223941 (64.6) | 22248 (70.6) |
| Charlson Comorbidity Index >1 (%) | | 5368 (49.9) | 22543 ( 6.5) | 2649 ( 8.4) |
| ISS (median [IQR]) | | 10 [8 , 20 ] | 10 [5 , 16 ] | 26 [22 , 33 ] |
| ISS > 15 (%) | | 4126 (38.3) | 87638 (25.3) | 29441 (93.4) |
| AIS Head > 3 (%) | | 833 ( 7.7) | 25089 ( 7.2) | 25024 (79.4) |
| AIS Thorax > 3 (%) | | 814 ( 7.6) | 10959 ( 3.2) | 3165 (10.0) |
| AIS Face > 3 (%) | | 17 ( 0.2) | 208 ( 0.1) | 219 ( 0.7) |
| AIS Abdomen > 3 (%) | | 1396 (13.0) | 7944 ( 2.3) | 934 ( 3.0) |
| AIS Spine > 3 (%) | | 467 ( 4.3) | 5271 ( 1.5) | 906 ( 2.9) |
| AIS Extremity > 3 (%) | | 273 ( 2.5) | 3057 ( 0.9) | 680 ( 2.2) |
| AIS Other > 3 (%) | | 89 ( 0.8) | 654 ( 0.2) | 57 ( 0.2) |
|  |  | | | |
| Oxygen saturation (median [IQR]) | | 96 [92 , 98 ] | 98 [96 , 99 ] | 96 [91 , 98 ] |
| Respiration Rate (median [IQR]) | | 18 [16 , 20 ] | 18 [16 , 20 ] | 16 [12 , 20 ] |
| Pulse (median [IQR]) | | 88 [74 , 105 ] | 89 [77 , 102 ] | 88 [73 , 107 ] |
| SBP (median [IQR]) | | 165 [140 , 190 ] | 138 [120 , 155 ] | 124 [100 , 142 ] |
| SBP < 90 (%) | | 119 ( 1.1) | 11447 ( 3.3) | 5179 (16.4) |
| GCS (median [IQR]) | | 15 [9 , 15 ] | 15 [15 , 15 ] | 7 [3 , 14 ] |
| GCS < 8 (%) | | 2571 (23.9) | 10380 ( 3.0) | 17251 (54.7) |
| Intubated (%) | | 875 ( 8.1) | 11266 ( 3.3) | 4120 (13.1) |
| Travel time to first hospital (median [IQR]) | | 52 [38 , 76 ] | 49 [35 , 71 ] | 49 [33 , 77 ] |
|  |  | | | |
| Transferred from other hospital (%) | | 4073 (37.8) | 110335 (31.8) | 11055 (35.1) |
| Level trauma center (%) | |  |  |  |
| 1 | | 6177 (57.4) | 200670 (57.9) | 19793 (62.8) |
| 2 | | 3788 (35.2) | 123246 (35.6) | 10364 (32.9) |
| 3 | | 799 ( 7.4) | 22648 ( 6.5) | 1360 ( 4.3) |
| Length of stay (median [IQR]) | | 6 [3 , 11 ] | 4 [2 , 7 ] | 7 [3 , 17 ] |
| In-hospital mortality (%) | | 2139 (19.9) | 9456 ( 2.7) | 11156 (35.4) |

Table S3: Characteristics of patients who substantially benefit from transportation to a Level-1 trauma centers, based on predicted benefit above 0 in 95% of bootstrapped samples from the regression model with interaction terms.

|  | No substantial benefit | Substantial benefit |
| --- | --- | --- |
| N | 297,087 | 91,758 |
| Age (median [IQR]) | 55 [35, 71] | 46 [29, 63] |
| Male (%) | 189704 (63.9) | 63225 (68.9) |
| Charlson Comorbidity Index >1 (%) | 29673 (10.0) | 887 ( 1.0) |
| ISS (median [IQR]) | 9 [5, 14] | 17 [13, 26] |
| ISS > 15 (%) | 61113 (20.6) | 60092 (65.5) |
| AIS Head > 3 (%) | 14535 ( 4.9) | 36411 (39.7) |
| AIS Thorax > 3 (%) | 8877 ( 3.0) | 6061 ( 6.6) |
| AIS Face > 3 (%) | 185 ( 0.1) | 259 ( 0.3) |
| AIS Abdomen > 3 (%) | 7432 ( 2.5) | 2842 ( 3.1) |
| AIS Spine > 3 (%) | 4952 ( 1.7) | 1692 ( 1.8) |
| AIS Extremity > 3 (%) | 2552 ( 0.9) | 1458 ( 1.6) |
| AIS Other > 3 (%) | 749 ( 0.3) | 51 ( 0.1) |
| **Prehospital features** | | |
| Oxygen saturation (median [IQR]) | 98 [95, 99] | 97 [95, 99] |
| Respiration Rate (median [IQR]) | 18 [16, 20] | 18 [16, 20] |
| Pulse (median [IQR]) | 88 [77, 102] | 90 [77, 105] |
| SBP (median [IQR]) | 141 [124, 160] | 122 [106, 138] |
| SBP < 90 (%) | 6713 ( 2.3) | 10032 (10.9) |
| GCS (median [IQR]) | 15 [15, 15] | 15 [11, 15] |
| GCS < 8 (%) | 10877 ( 3.7) | 19325 (21.1) |
| Intubated (%) | 9254 ( 3.1) | 7007 ( 7.6) |
| Travel time to first hospital (median [IQR]) | 49 [36, 71] | 48 [34, 72] |
| **Outcome measures** | | |
| Transferred from other hospital (%) | 96970 (32.6) | 28493 (31.1) |
| Level trauma center (%) |  |  |
| 1 | 170155 (57.3) | 56485 (61.6) |
| 2 | 106306 (35.8) | 31092 (33.9) |
| 3 | 20626 ( 6.9) | 4181 ( 4.6) |
| Length of stay (median [IQR]) | 4 [2, 7] | 5 [2, 11] |
| In-hospital mortality (%) | 11155 ( 3.8) | 11596 (12.6) |
