# Supplementary figures and images for: Identifying trauma patients with benefit from direct transportation to Level-1 trauma centers
Source: BMC Emerg Med. 2021 Aug 6;21:93. doi: 10.1186/s12873-021-00487-3 (PMC8344140; doi:10.1186/s12873-021-00487-3)

# Calibration benefit without interaction terms

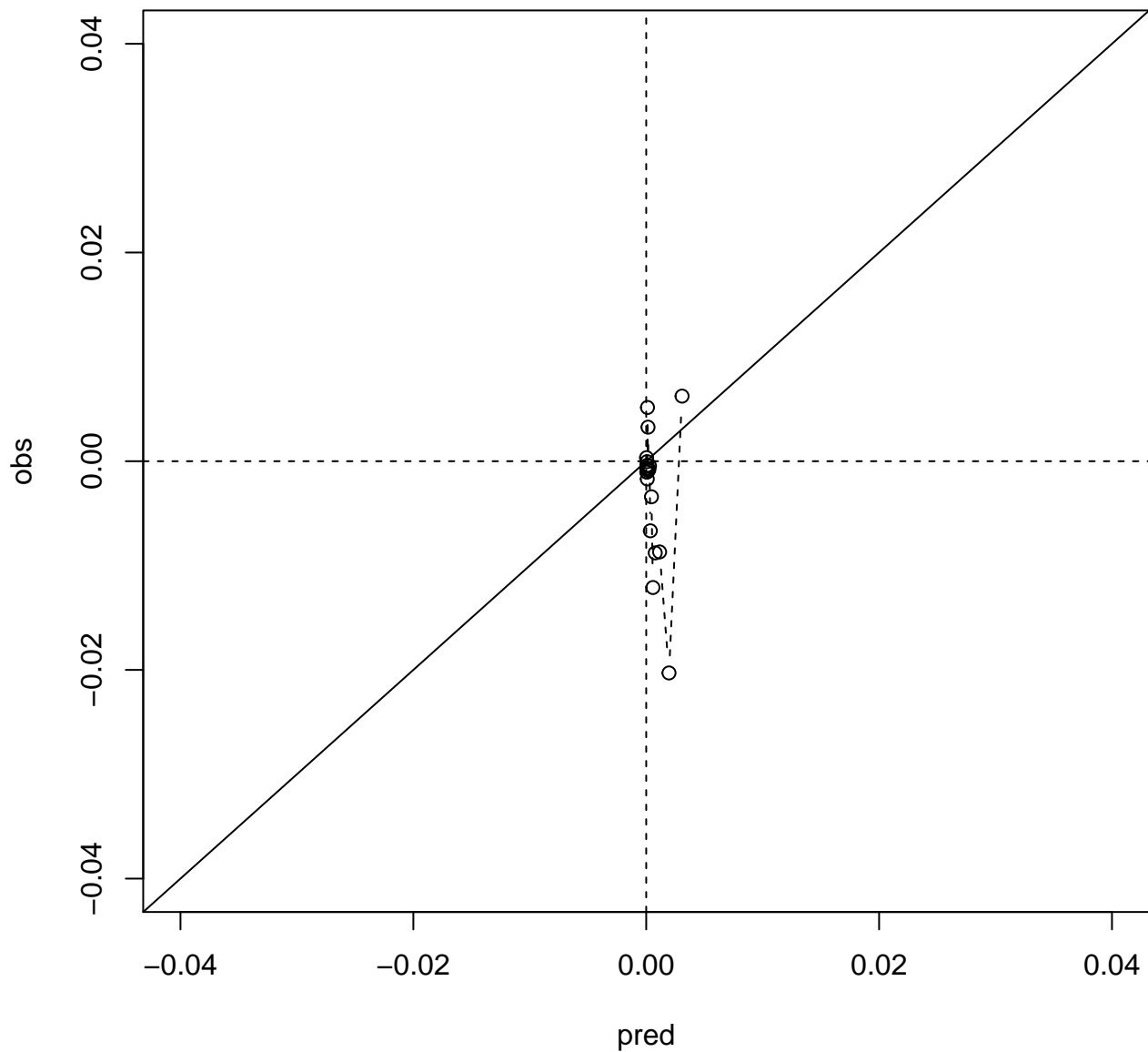

# Calibration benefit with interaction terms

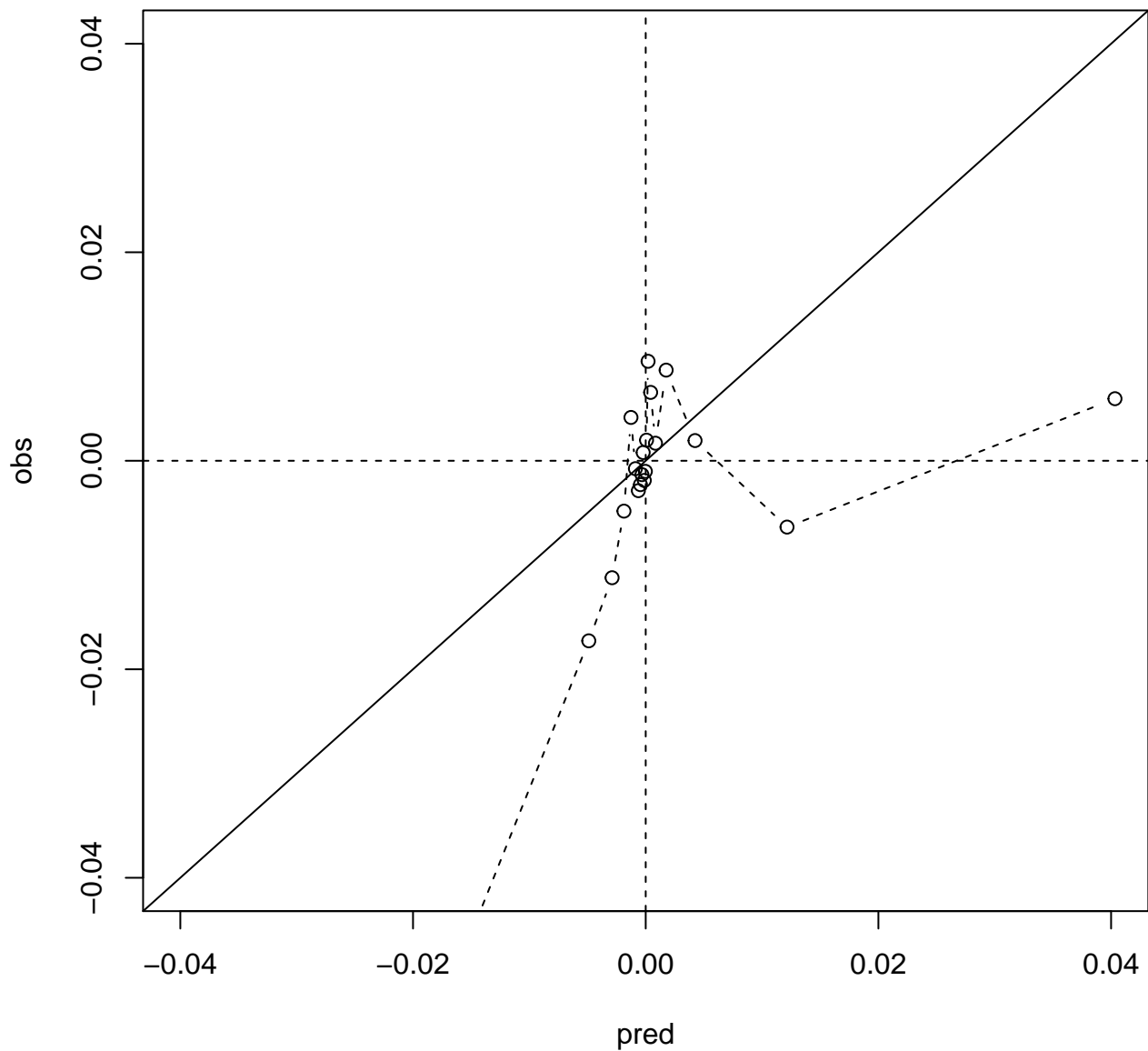

Supplement: Supplementary file 2 — Additional file 2: Figure S1. Calibration of benefit, for both the model with (B) and without interaction terms (A). Calibration of benefit is plotted with the predicted absolute treatment benefit on the x-axis and observed absolute treatment benefit on the y-axis. The study population was therefore divided in 20 equal groups based on the predicted absolute treatment benefit. A predicted absolute benefit of 1% (0.01) for a particular patient means 1% less mortality when this particular patient is brought to a Level-1/2 trauma center instead of a Level-3 trauma center. [file 12873_2021_487_MOESM2_ESM.pdf]

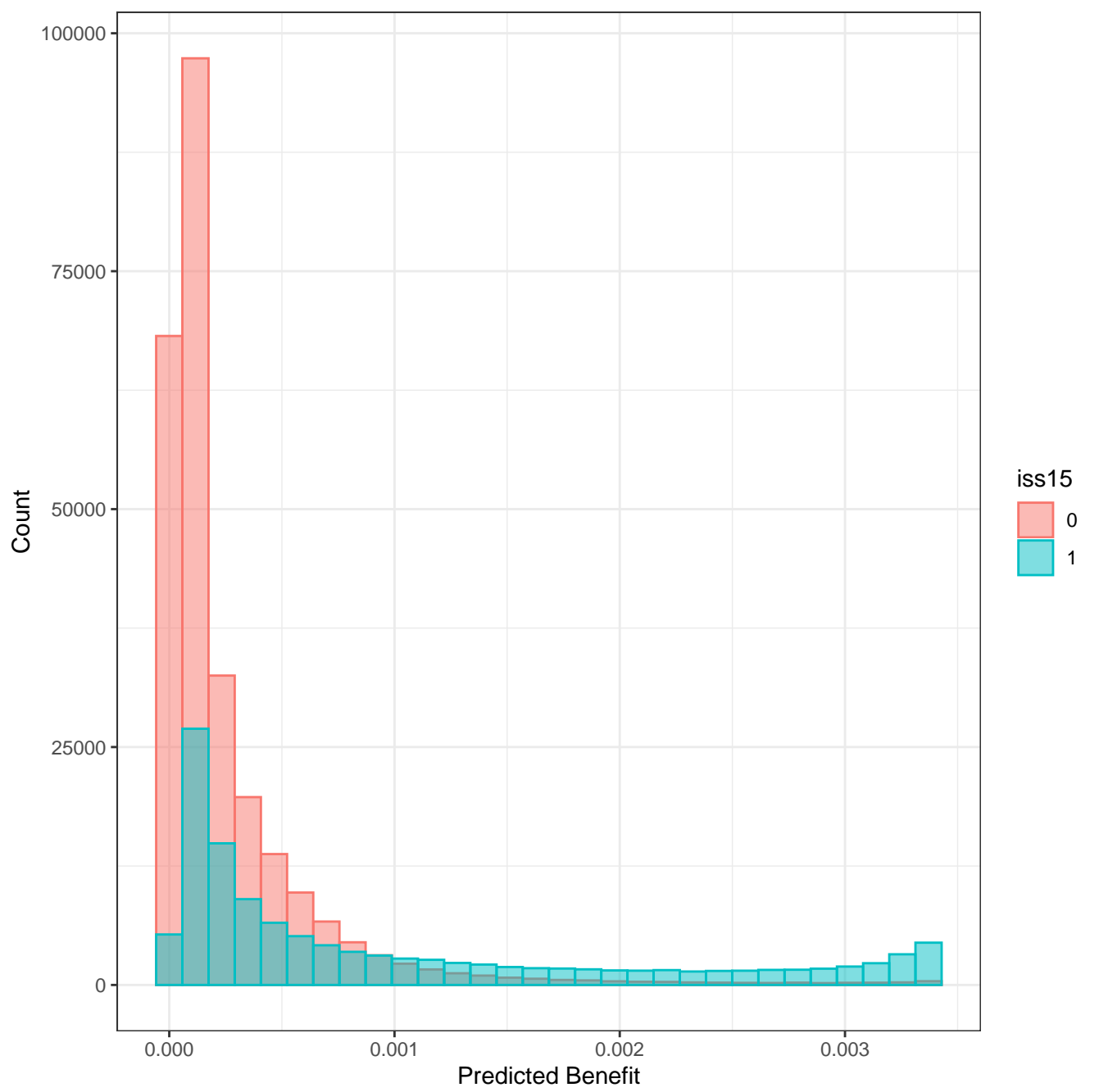

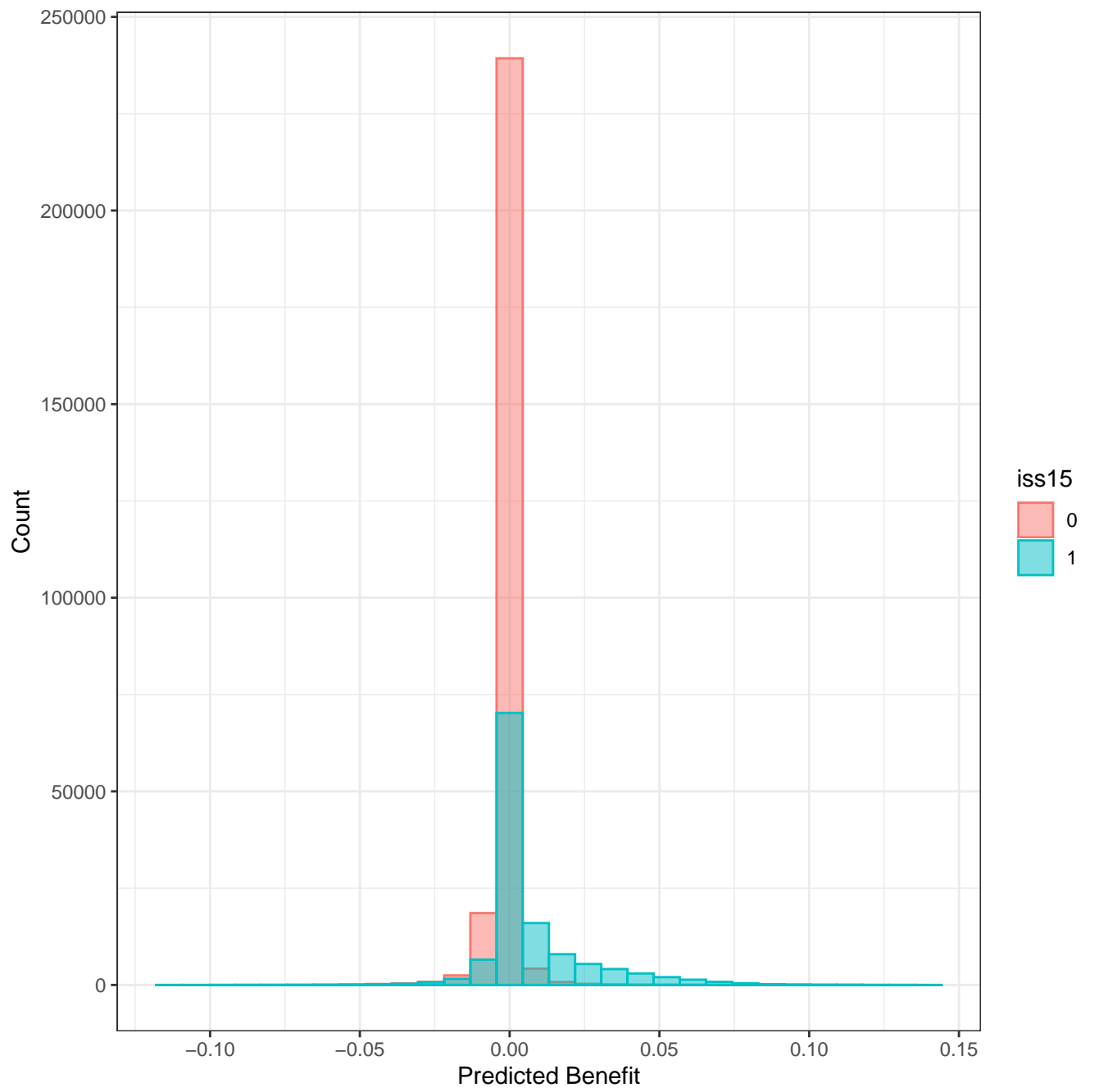

Supplement: Supplementary file 3 — Additional file 3: Figure S2. Histograms of the range of absolute benefit, for both the model with (B) and without interaction terms (A). Both histograms show the difference in absolute benefit for patients with ISS above 15 and patients with ISS below 15. A predicted absolute benefit of 1% (0.01) for a particular patient means 1% less mortality when this particular patient is brought to a Level-1/2 trauma center instead of a Level-3 trauma center. [file 12873_2021_487_MOESM3_ESM.pdf]

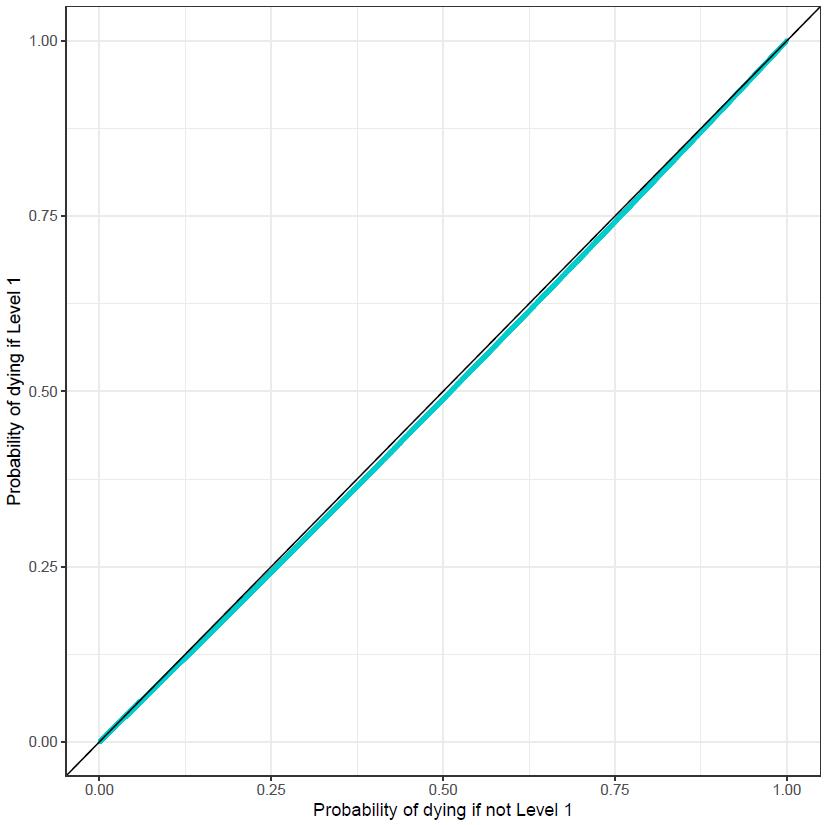

Supplement: Supplementary file 4 — Additional file 4: Figure S3. Patients who benefit with predicted benefit above 0 in 95% of the predictions from transportation to a Level-1 trauma center, following the models without (A) and with interaction terms (B). [file 12873_2021_487_MOESM4_ESM.zip › eFigure 1AR2.jpg]

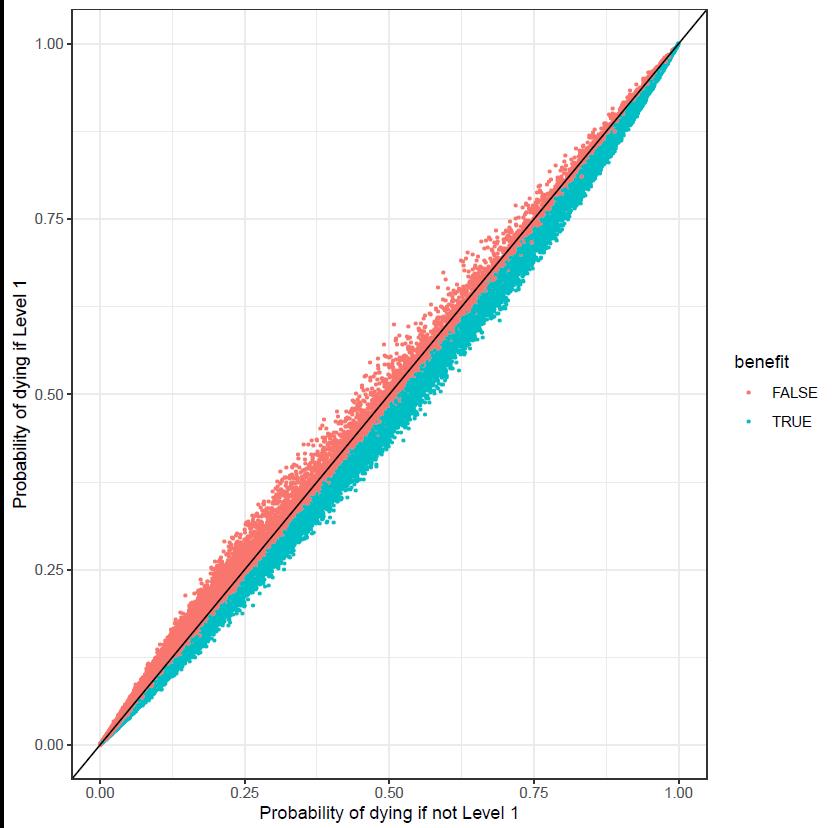

Supplement: Supplementary file 4 — Additional file 4: Figure S3. Patients who benefit with predicted benefit above 0 in 95% of the predictions from transportation to a Level-1 trauma center, following the models without (A) and with interaction terms (B). [file 12873_2021_487_MOESM4_ESM.zip › eFigure 1BR2.jpg]
